# Use of whole-genome sequencing to detect transmission of group A *Streptococcus* in Houston, TX

**DOI:** 10.1099/acmi.0.000351

**Published:** 2022-05-19

**Authors:** Zain I. Alamarat, Misu A. Sanson, J. Chase McNeil, Carol J. Baker, Anthony R. Flores

**Affiliations:** ^1^​ Division of Infectious Diseases, Department of Pediatrics, McGovern Medical School, University of Texas Health Sciences Center at Houston, Houston, TX, USA; ^2^​ Section of Infectious Diseases, Department of Pediatrics, Baylor College of Medicine and Texas Children’s Hospital, Houston, TX, USA; ^3^​ Center for Antimicrobial Resistance and Microbial Genomics, McGovern Medical School, University of Texas Health Sciences Center at Houston, Houston, TX, USA; ^†^​ Present address: Division of Infectious Diseases, Department of Pediatrics, College of Medicine, University of Arkansas for Medical Sciences, Little Rock, AR, USA

**Keywords:** group A streptococcus, transmission, invasive disease, whole-genome sequencing

## Abstract

We used a combination of local, comprehensive strain surveillance and bacterial whole-genome sequencing to identify potential transmission events of group A streptococcus (GAS) in Houston, TX, USA. We identified pharyngeal and skin and soft tissue sources of infection as having important roles in community GAS transmission, including invasive diseases.

## Data Summary

All raw genome sequences generated in this study are publicly available from the National Center for Biotechnology Information (NCBI) under BioProject accession number PRJNA728081.

## Introduction

Disease due to group A streptococcus (GAS) occurs frequently in children, usually manifesting as pharyngitis or superficial skin infections. However, invasive GAS (iGAS) infections, albeit less common (e.g. necrotizing fasciitis or streptococcal toxic shock syndrome), are responsible for significant morbidity and mortality. National-level surveillance at the Centers for Disease Control and Prevention (CDC) estimates more than 10 000 cases and approximately 1500 deaths due to iGAS annually in the USA [1]. More recently, rates of iGAS disease have increased from 3 to 4 cases per 100 000 population between 1997–2003 to >7 cases per 100 000 in 2018, and have been linked to injection drug use and homelessness [2].

Since the seminal studies by Hamburger in the 1950s [3], much interest surrounds the role of person-to-person GAS transmission in subsequent development of invasive disease. Further, there is increasing interest in detecting potential transmission events (PTEs) for GAS disease and in employing surveillance data to inform recommendations for chemoprophylaxis of close contacts [4]. Studies conducted by the CDC have demonstrated utility in epidemiological investigations that have shown an increase in secondary attack rate from 66.1 to 102 per 100 000 when comparing 2003 to 2019, a rate that is higher among older adults with co-morbidities [5, 6]. However, previous studies were limited to iGAS disease and did not include pharyngitis or superficial skin infections that are known to occur more frequently and are likely to contribute to overall disease transmission.

## Methods

We used an existing comprehensive GAS passive surveillance system approved by the Committee for the Protection of Human Subjects at UTHealth/McGovern Medical School and Baylor College of Medicine in Houston, TX, USA [7]. GAS isolates and associated metadata derived from pharyngeal (PHG), skin and soft tissue infection (SSTI) and invasive (INV) diseases in children (0–18 years of age) and adults between July 2017– December 2019 were included in the study. All isolates were *emm* typed as previously described [7]. PTEs were defined as GAS isolates of the same *emm* type, originating from the same zip code, and occurring within 30 days of each other. By default, index cases within index/PTE sets were defined temporally as occurring prior to any PTE. Bacterial whole-genome sequencing (WGS) was performed on an Illumina MiSeq instrument and processed to determine strain variation (single nucleotide polymorphisms, SNPs) as previously described [7]. Briefly, following on-instrument QC, short-read sequences were error-corrected using SPAdes (v 3.12.0) [8], mapped to an *emm* type-specific reference genome [MGAS2221 (*emm1*), CP043530; MGAS315 (*emm3*), AE014074; ABC208 (*emm4*), CP049690; MGAS10394 (*emm6*), CP000003; MGAS2096 (*emm12*), CP000261; and MGAS6180 (*emm28*), CP000056] using SMALT (v 0.7.6) (https://www.sanger.ac.uk/science/tools/smalt-0), and polymorphisms were identified using freebayes (v 1.2.0) (https://github.com/freebayes/freebayes). SNP distances excluded known mobile genetic elements (e.g. prophage, integrative conjugative elements). Based on studies in GAS and *Staphylococcus aureus* [9, 10]*,* we used a strict cutoff of <15 SNPs (core genome only) in support of person-to-person transmission. All bacterial whole-genome sequence data generated were been deposited under BioProject number PRJNA728081.

## Results

A total of 1290 isolates were included in the study, of which 171 (13.3 %) and 1119 (86.7 %) were collected from adults and children, respectively (Table 1). The majority (880/1290, 69 %) of GAS isolates were derived from pharyngeal infections. The most common *emm* types by frequency were *emm1* (21.3 %), *emm12* (19.8 %), *emm89* (8.1 %), *emm28* (7.5 %) and *emm6* (6.4 %). Using the defined criteria, 94 PTEs were identified, originating from 74 index cases. Index cases most frequently resulted in a single PTEs (*n*=74). However, 16 PTE clusters (≥2 events meeting the defined PTE criteria) were also identified. Overall, the average number of PTEs per index case was 1.3 (range: 1–3). Of the index cases, 10 GAS isolates were derived from iGAS disease (10/74, 13.5 %), 6 from SSTI (6/74, 8.1%) and the remainder (58/74, 78.3 %) from pharyngeal infections. Index GAS isolates, and therefore PTEs, were most likely to be either *emm1* (76/168, 45.2 %) or *emm12* (51/168, 30.4 %).

**Table 1.** Characteristics of GAS surveillance population and PTE

Surveillance population characteristics					
Disease*	**Total (%**)	**Adult (%**)	**Paediatric (%**)	***emm*†**	
**INV**	221 (17)	52 (23.5)	169 (76.5)	1, 12, 89, 6, 4	
**SSTI**	180 (14)	47 (26.1)	133 (73.9)	1, 12, 4, 89, 92	
**PHG**	889 (69)	72 (8.1)	817 (91.9)	1, 12, 89, 28, 6	
**Total**	1290	171 (13.3)	1119 (86.7)	1, 12, 89, 28, 6	
**Potential transmission events**					
**PTE‡**	**Age (years)§**	**Days||**	**Disease¶**	***emm***	SNPs**
**17**	2/65	7	INV/INV	1	1
**20**	6/3	29	INV/PHG	12	3
**28**	16/7	5	PHG/SSTI	1	14
**36**	3/33	18	PHG/SSTI	1	10
**42**	5/8	1	INV/PHG	1	0
**45††**	6/6	8	PHG/PHG	1	13

*Disease category as defined in text.

†Most common *emm* types in order of frequency for given disease type.

‡Potential transmission event (single index to PTE) as defined in Table S1.

§Age of index/transmission subjects in years.

||Number of days between index case diagnosis and PTE.

¶Disease type for index/transmission.

**Number of SNPs in core genome differentiating PTE from index isolate.

††WGS evidence for transmission between PTE-1 and PTE-2 of a four-strain cluster as shown in Table S1.

INV, invasive; PHG, pharyngeal; PTE, potential transmission event; SNP, single nucleotide polymorphism; SSTI, skin and soft tissue infection.

A substantial proportion of PTEs resulted in SSTIs (10/94, 10.6 %) and iGAS infections (10/94, 10.5 %). The four most common GAS *emm* types identified among PTEs were *emm1* (43/94, 45.7 %), *emm12*(30/94, 31.9 %), *emm4* (6/94, 6.4 %) and *emm6* (5/94, 5.3%). Not surprisingly given the paediatric bias in our surveillance, index and PTE isolates were derived from patients with a mean age of 9.35 (range 0.5–49.5 years) and 12.50 (range 0.22–71.1 years), respectively. The median interval between the index and secondary cases was 8 days (range 0–30 days).

Previous studies examining surveillance-based GAS transmission included only invasive disease and included direct interview of index cases to facilitate linking PTEs [5, 6]. In the absence of index case interviews, we chose to confirm PTEs using bacterial WGS. Further, WGS was only performed for events (index and PTE) that included either an SSTI or invasive infection, as these may more likely result in the need for antimicrobial prophylaxis. Complete genome sequence was obtained from a total of 61 GAS strains derived from 28 events, including 4 PTE clusters (Table S1). Sequenced strains included *emm1* (*n*=36), *emm12* (*n*=10), *emm6* (*n*=6), *emm28* (*n*=5), *emm3* (*n*=2) and *emm4* (*n*=2). WGS supported transmission in 6 (21 % of PTEs for which WGS was available) independent events (Table 1 and S1). Importantly, the combination of comprehensive surveillance (i.e. invasive and non-invasive infection types) and bacterial WGS demonstrates pharyngeal infection as a key factor in person-to-person transmission.

## Discussion

Severe GAS infection may be associated with high morbidity and mortality, especially in vulnerable populations such as the elderly and those with co-morbidities [1]. In addition, GAS is known to spread easily person to person, increasing the risk of infection and disease in close contacts [3]. Expert opinion is divided regarding recommendations to provide antimicrobial prophylaxis to close contacts of severe iGAS disease [4]. Using national-level invasive GAS disease surveillance, the CDC has identified secondary cases of GAS infection and reported that such cases were more likely to occur in those 65 years of age [5]. However, non-invasive GAS disease (e.g. pharyngeal and SSTI) is not included in the CDC surveillance – infections that are likely to be a critical component in community spread of GAS and may lead to invasive disease. In this study, we used established, comprehensive local surveillance in a large metropolitan area (Houston, TX, USA) to retrospectively identify potential transmission events over an 18-month period. In place of in-person interviews to determine possible transmission to close contacts, we used bacterial WGS as evidence of transmission events. Our approach identified several PTEs, with only one involving both index and transmission isolates derived from invasive disease. Inasmuch as non-invasive GAS infection has been temporally associated with invasive disease outbreaks [11, 12], inclusion of pharyngeal (carriage and pharyngitis) and SSTI GAS surveillance may serve to enhance identification of community transmission and possible outbreak detection. Thus, our data support continued comprehensive surveillance combined with near real-time bacterial WGS as a powerful tool to rapidly identify GAS person-to-person transmission, potentially facilitating provider decision-making in antimicrobial prophylaxis against invasive disease.

Previous studies have used genomic SNP-based phylogeny to infer transmission, but these have been limited to outbreaks within hospital units/wards or group residential facilities [9, 13]. The study by Coelho *et al*. examined multiple GAS outbreaks in England, demonstrating the utility of WGS in outbreak investigation [9]. Importantly, that study demonstrated variation in outbreak-related strain SNP distance dependent, in part, upon *emm* type. Specifically, *emm1* outbreak isolates showed a mean SNP distance of 0.5, which was much lower than that in non-outbreak *emm1* strains (mean SNP distance: 28.6) [9]. We observed similar findings in PTEs involving *emm1* GAS. While our mean SNP distance for *emm1* PTEs using the strict cutoff criterion of 15 SNPs (core genome) was higher at 7.6, it was substantially lower than that in the *emm1* population as a whole (30.2, 99 % CI: 23.6–36.6). For the single *emm12* PTE, the SNP difference of 3 (Table 1) was also substantially lower than the mean SNP distance for the *emm12* population sequenced (84.7, 99 % CI: 39.1–130.3). The use of more stringent criteria (e.g. SNP distance <10) would not alter the conclusions and as a first-pass more liberal cutoffs would be preferred to ensure capture of PTEs in routine surveillance.

A few limitations of our study need to be mentioned. Our study was retrospective, therefore not allowing in-person interviews or swabbing of close contacts to more thoroughly examine transmission or secondary attack rates. The use of patient home residence zip code as one of the criteria is likely to have reduced the number of PTEs identified, given that individuals are likely to have several contacts outside of this area in a large metropolitan setting.

## Conclusions

The combination of comprehensive GAS disease surveillance and strain WGS is a useful tool in the identification of person-to-person transmission events. Expanded efforts including near real-time WGS may facilitate detection of outbreaks and in particular emergence of novel clones. Our study serves as a model of bacterial disease surveillance and may find utility in community and hospital-based settings for several important pathogens.

## Supplementary Data

Supplementary material 1
